# Mitigating Monomer Leaching and Resin-Related Hypersensitivity in Removable Orthodontics Through Bio-Inspired Surface Modifications: A Narrative Review

**DOI:** 10.3390/biomimetics11080555

**Published:** 2026-08-05

**Authors:** Lucia Giannini, Marco Farronato, Antonino Manti, Cinzia Maspero

**Affiliations:** 1 Fondazione IRCCS Cà Granda Ospedale Maggiore Policlinico, 20122 Milan, Italy; dott.lucia.giannini@gmail.com (L.G.); marco.farronato@unimi.it (M.F.); cinzia.maspero@unimi.it (C.M.); 2 Department of Biomedical, Surgical and Dental Sciences, School of Dentistry, University of Milan, 20122 Milan, Italy

**Keywords:** bio-inspired coatings, removable orthodontic appliances, polymethyl methacrylate, monomer leaching, hypersensitivity, titanium dioxide, biofilm

## Abstract

Background: During clinical service, removable orthodontic appliances are continuously exposed to environmental challenges that may compromise material stability and contribute to potentially increasing the risk of local inflammatory responses and hypersensitivity reactions. Method: A literature search was conducted using PubMed/MEDLINE, Scopus, and Web of Science databases. Studies investigating bio-inspired and surface-engineering approaches applicable to orthodontic polymers, acrylic resins, aligner materials, and related dental biomaterials were considered. Evidence from orthodontic adhesives and non-bio-inspired barrier coatings was included when relevant as comparative or translational support. Particular attention was given to polydopamine coatings, polyphenol- and tannic acid-based coatings, chitosan systems, peptide-functionalized surfaces, titanium and titanium oxide-based modifications, nanoparticle-enriched coatings, biomimetic hydrogel-like barriers, and conventional barrier coatings used as comparators. Results: Available evidence suggests that surface modifications reduce monomer diffusion, bacterial adhesion, biofilm formation, and improve cellular responses. Bio-inspired and titanium-based coatings show particular promise by enhancing biocompatibility while providing antimicrobial, antioxidant, anti-inflammatory, and protective barrier properties. Conclusions Bio-inspired surface engineering is a promising approach to improve the biological safety of removable orthodontic appliances. However, further standardized in vitro, in situ, and clinical studies are needed to confirm their long-term stability, durability, and clinical effectiveness in reducing hypersensitivity and other adverse biological effects.

## 1. Introduction

Removable orthodontic appliances remain widely used despite the growing diffusion of clear aligners and digitally manufactured devices. Conventional acrylic-based appliances are still central in interceptive orthodontics, retention, functional orthopedic treatment, and removable expansion, including polymethyl methacrylate (PMMA) plates, Hawley retainers, functional appliances, removable expanders, clear thermoplastic aligners, and hybrid devices combining acrylic resin with wires, screws, or springs. Fixed therapy also uses adhesives and resin-based bonding materials, adding exposure to methacrylate-derived compounds [1,2].

Biological safety is especially relevant in children and adolescents, who may wear such appliances for months or, most commonly, years. Prolonged intraoral exposure increases cumulative biomaterial–tissue interactions and concerns about chronic low-dose chemical release. In parallel, titanium and titanium alloys have been proposed as biocompatible alternatives to nickel-containing alloys in susceptible patients, while digital workflows and additive manufacturing have expanded patient-specific titanium orthodontic devices [3,4].

The oral cavity is a dynamic environment in which salivary enzymes, pH fluctuations, thermal changes, mechanical loading, and microbial metabolism may accelerate material aging and degradation. Because complete polymerization is rarely achieved, methacrylate-based polymers may release residual monomers and degradation products over time. The extent of leaching depends on material composition, manufacturing procedures, surface characteristics, environmental conditions, and exposure duration [5].

Material-derived compounds have been widely investigated in restorative dentistry and orthodontics. Residual monomers and degradation products may induce effects ranging from transient irritation to complex cellular responses [6,7]. Local manifestations include mucosal irritation, erythema, contact stomatitis, burning sensations, oral discomfort, and delayed hypersensitivity. Experimental studies have associated methacrylate monomers with oxidative stress, inflammatory activation, mitochondrial dysfunction, apoptosis, and reduced viability in oral keratinocytes and fibroblasts. Beyond cytotoxicity, genotoxic and mutagenic potential is gaining attention. Experimental evidence suggests that DNA damage related to reactive oxygen species, oxidative stress pathways, and electrophilic interactions may occur following exposure to several methacrylate-derived compounds. Although in vitro findings cannot be directly translated into clinical risk, they support broader evaluation including oxidative stress, genotoxicity, and mutagenicity [8,9].

Biofilm formation is another mechanism through which appliances may affect oral health [10,11]. Experimental studies indicate that surface characteristics influence bacterial adhesion and biofilm formation, which may contribute to oral dysbiosis, mucosal inflammation, and enamel demineralization. Consequently, reducing bacterial adhesion represents an additional objective of advanced surface-engineering strategies. Thus, reducing bacterial adhesion is an important objective alongside minimizing monomer release [12].

Strategies to reduce residual monomer release include post-polymerization heat treatments, prolonged water storage, additional curing, polishing, and washing before clinical delivery. These procedures may reduce extractable compounds by improving conversion or promoting monomer diffusion before insertion, but they do not eliminate residual substances [13]. They also do not fully address long-term surface degradation, biofilm accumulation, or chronic biomaterial–tissue interactions; therefore, concerns may persist despite optimized protocols [12,13,14,15].

Recent biomaterials research has shifted from bulk composition toward surface-interface engineering. Surface functionalization aims to create a biologically favorable oral interface, influencing bacterial adhesion, protein adsorption, inflammatory responses, cellular behavior, and molecular diffusion without substantially altering mechanical properties. Bio-inspired strategies draw from natural systems, including mussel adhesion, plant-derived polyphenols, antimicrobial peptides, mucosal barriers, and extracellular matrix interactions. Engineered coatings may act as diffusion barriers, antimicrobial platforms, anti-inflammatory interfaces, or multifunctional bioactive surfaces. Accordingly, bio-inspired surface engineering may improve appliance safety while preserving clinical performance. From a biomimetic perspective, these coatings are engineered analogs of natural interfaces, such as mussel-inspired adhesive films, plant-derived antioxidant barriers, polysaccharide antimicrobial matrices, peptide-mediated recognition systems, hydrated mucosa-like surfaces, and oxide-stabilized mineral interfaces.

Despite the growing interest in biomimetic surface engineering across dentistry, no previous narrative review has specifically focused on bio-inspired surface modifications aimed at reducing monomer leaching and resin-related hypersensitivity in removable orthodontic appliances. Existing reviews mainly address antimicrobial coatings, restorative biomaterials, or implant surfaces without integrating biological safety, monomer diffusion, oxidative stress, biofilm formation, and translational applicability within the specific context of removable orthodontics. Therefore, an important knowledge gap still exists regarding the potential role of biomimetic surface engineering in improving the biological safety of removable orthodontic appliances.

The aim of this review is to critically evaluate bio-inspired and biomimetic surface modification strategies that may reduce monomer release, may improve biocompatibility, limit bacterial adhesion, and mitigate resin-related inflammatory and hypersensitivity reactions associated with removable orthodontic appliances.

The review additionally discusses whether these strategies have the potential to reduce cytotoxicity, genotoxicity, and oxidative stress, decrease bacterial adhesion and biofilm formation, improve surface wettability and reduce roughness, preserve transparency, elasticity, mechanical strength, and dimensional stability, remain stable under brushing, salivary exposure, pH fluctuations, and thermal cycling, and are feasible for clinical or industrial implementation.

## 2. Materials and Methods

### 2.1. Study Design and Review Approach

This manuscript was designed as a comprehensive narrative literature review aimed at synthesizing current evidence regarding bio-inspired and biomimetic surface engineering approaches applicable to removable orthodontic biomaterials. Because the available evidence is heterogeneous and largely preclinical, the review emphasizes mechanistic interpretation, translational relevance, current gaps and future research priorities rather than quantitative meta-analysis.

### 2.2. Databases

The literature search was conducted using PubMed/MEDLINE, Scopus, and Web of Science. The search was last updated on 1 April 2026 and primarily considered publications issued between January 2000 and April 2026. Earlier seminal studies identified through manual reference screening were retained when considered necessary to provide historical or mechanistic context. The reference lists of relevant original articles and reviews were also manually screened to identify additional potentially eligible studies.

### 2.3. Search Strategy

The search strategy combined controlled vocabulary and free-text terms related to orthodontic materials, monomer release, biological safety, titanium-based materials, biomimetic interfaces, and surface engineering technologies.

The search strategy included the following keywords and keyword combinations: “orthodontic resin”, “PMMA”, “removable orthodontic appliance”, “aligner material”, “monomer leaching”, “residual monomer”, “methyl methacrylate”, “bisphenol A glycidyl methacrylate (Bis-GMA)”, “cytotoxicity”, “genotoxicity”, “Ames test”, “allergic reaction”, “contact stomatitis”, “bio-inspired coating”, “polydopamine”, “mussel-inspired coating”, “tannic acid”, “polyphenol coating”, “chitosan coating”, “peptide coating”, “titanium oxide”, “antibacterial coating”, “orthodontic appliance surface modification”, “titanium in orthodontics”, “orthodontic appliances in titanium”, and “allergic patients”.

The search covered evidence concerning removable orthodontic appliances, orthodontic and dental polymers, residual monomer release, biological safety, hypersensitivity, cytotoxicity, genotoxicity, mutagenicity, bacterial adhesion, biofilm formation, surface properties, coating durability, and bio-inspired or biomimetic surface modification strategies. Evidence derived from restorative dentistry, prosthodontics, implantology, and general biomaterials research was considered only when it provided relevant translational support for removable orthodontic applications.

### 2.4. Inclusion Criteria

Studies were considered eligible when they met one or more of the following criteria: in vitro investigations, in situ studies, clinical studies, investigations involving orthodontic resins, dental PMMA, aligner polymers, retainers, adhesives, or related methacrylate-based materials, studies evaluating monomer release, cytotoxicity, genotoxicity, hypersensitivity, bacterial adhesion, surface roughness, wettability, or coating stability and studies investigating bio-inspired or biomimetic coatings applicable to dental polymers.

### 2.5. Exclusion Criteria

The following studies were excluded: investigations unrelated to dental or orthodontic materials, studies lacking biological or surface-related outcomes, exclusively mechanical studies without relevance to biocompatibility or leaching phenomena, narrative reviews and systematic reviews, case reports not specifically addressing orthodontic material hypersensitivity and articles unavailable in English.

### 2.6. Narrative Synthesis and Biomimetic Categorization

Because this study was designed as a narrative review, no formal risk-of-bias assessment tool was applied. The relevance and methodological quality of the included evidence were appraised qualitatively according to study design, clarity of the experimental methodology, appropriateness of the investigated materials and biological models, relevance of the evaluated outcomes to the review question, directness of the evidence to removable orthodontic appliances, consistency of the findings, and translational applicability. Evidence obtained directly from removable orthodontic materials was distinguished from indirect evidence extrapolated from other dental or biomedical fields.

## 3. Results

### 3.1. Orthodontic Resins and Monomer Release

Methacrylate-based polymers remain the most widely used materials in removable orthodontics. PMMA continues to be extensively employed in removable functional appliances, retainers and removable expansion devices because of its favorable mechanical properties, low manufacturing cost, and clinical versatility. Similarly, resin-based adhesives and composite materials are routinely used for bonding procedures, while thermoplastic polymers constitute the basis of modern aligner systems [16,17].

Despite their widespread clinical success, these materials are not chemically inert. Residual monomers may remain trapped within the polymer matrix following polymerization and may subsequently diffuse into the oral environment. The extent of monomer release depends on multiple variables, including polymerization efficiency, degree of conversion, material composition, storage conditions, water sorption, surface finishing and long-term degradation. The characteristics of the included studies are summarized in Table 1.

#### 3.1.1. PMMA and Methyl Methacrylate Release

Residual methyl methacrylate (MMA) represents one of the most extensively investigated compounds in removable orthodontics. Although modern processing techniques have significantly reduced residual monomer content, complete conversion of MMA into polymerized PMMA is rarely achieved.

Several studies have demonstrated that MMA release is greatest during the first hours or days following fabrication but may continue at lower levels over prolonged periods. Water absorption promotes polymer swelling and facilitates diffusion of residual compounds toward the material surface. In addition, mechanical wear and enzymatic degradation may progressively expose previously entrapped molecules.

#### 3.1.2. Bis-GMA-Based Orthodontic Adhesives

Orthodontic bonding systems frequently contain Bis-GMA and related dimethacrylates. These materials may release residual monomers, particularly when polymerization is incomplete or when aging processes compromise polymer integrity. Although released concentrations are generally low, concern persists regarding potential biological effects because exposure occurs in close proximity to oral soft tissues.

#### 3.1.3. TEGDMA, UDMA, HEMA and Related Monomers

Triethylene glycol dimethacrylate (TEGDMA), urethane dimethacrylate (UDMA), and 2-hydroxyethyl methacrylate (HEMA) are commonly incorporated into resin formulations as diluent monomers or modifiers. Compared with larger molecules such as Bis-GMA, these monomers often exhibit greater mobility and may diffuse more readily through polymer networks. Experimental investigations have demonstrated their capacity to induce oxidative stress, alter cellular metabolism, and influence inflammatory signaling pathways under laboratory conditions.

#### 3.1.4. Degradation Products

In addition to residual monomers, aging processes generate secondary degradation products. Hydrolysis, enzymatic activity, oxidation, and mechanical wear may produce low-molecular-weight compounds capable of interacting with oral tissues. These degradation products may contribute to cumulative biological effects, even when initial residual monomer concentrations are relatively low.

#### 3.1.5. Influence of Oral Environmental Factors

The oral environment strongly influences leaching behavior. Saliva composition, pH fluctuations, thermal cycling, microbial metabolism, and mechanical abrasion can all accelerate degradation processes. Acidic conditions may increase hydrolytic degradation, while repeated thermal stresses can induce microstructural changes within polymers. Similarly, brushing procedures and mastication may increase surface roughness, facilitating both compound release and bacterial colonization.

#### 3.1.6. Metallic Appliances and Titanium-Based Alternatives

Conventional orthodontic appliances often contain stainless steel and nickel–titanium alloys. Corrosion processes may result in the release of nickel, chromium, cobalt, and other metallic ions. In contrast, titanium and titanium alloys exhibit superior corrosion resistance due to the formation of a stable oxide layer. Consequently, titanium-based devices are increasingly considered attractive alternatives for patients with known metal hypersensitivity or concerns regarding long-term ion release.

### 3.2. Cytotoxicity, Genotoxicity and Mutagenicity

The biological effects of orthodontic materials extend beyond simple tissue irritation. Increasing evidence suggests that residual monomers may influence cellular function through multiple mechanisms involving oxidative stress, inflammatory activation, DNA damage, and altered cell viability. Human gingival fibroblasts and oral keratinocytes are commonly used to evaluate material biocompatibility. Numerous investigations have demonstrated that methacrylate monomers can reduce cell viability in a dose-dependent manner. Observed effects include decreased mitochondrial activity, altered cellular proliferation, membrane damage, apoptosis induction, and impaired wound healing responses. The severity of these effects depends on monomer concentration, exposure duration, and cell type.

Oxidative stress is considered one of the principal mechanisms underlying methacrylate-associated toxicity. Reactive oxygen species generated following monomer exposure may damage proteins, lipids, and nucleic acids. Experimental studies have reported increased oxidative stress markers, mitochondrial dysfunction, and DNA strand breaks following exposure to methacrylate-derived compounds.

Growing attention has focused on mutagenicity assessment as a complementary endpoint for biological safety evaluation. The Ames test remains one of the most widely used screening tools for detecting mutagenic potential. Recent investigations evaluating eluates derived from dental and orthodontic materials have emphasized the importance of assessing mutagenicity under both metabolically activated and non-activated conditions. Such approaches better reflect the potential transformation of released compounds into biologically active metabolites. The incorporation of Ames testing into orthodontic biomaterial assessment broadens the understanding of biological risk beyond conventional cytotoxicity measurements. A recent study specifically investigating orthodontic resins by means of the Ames test demonstrated that some commercially available resin systems and/or their base components induced significant mutagenic activity in Salmonella typhimurium strains TA100 and TA1535, with increases in revertant colonies of up to approximately 145% compared with controls, whereas no significant effects were observed in TA98, TA1537, or TA1538. These findings reinforce the importance of extending biomaterial safety assessment beyond cytotoxicity alone and support the inclusion of mutagenicity testing among the biological endpoints relevant to removable orthodontic materials [9,18].

### 3.3. Resin-Related Inflammatory and Hypersensitivity Reactions

The term “micro-allergy” lacks a universally accepted clinical definition. More appropriately, adverse reactions associated with orthodontic resins may be described as low-grade mucosal hypersensitivity or resin-related inflammatory and hypersensitivity reactions.

Delayed-type hypersensitivity represents the most frequently reported immunological mechanism associated with methacrylate exposure. Residual monomers may act as haptens capable of binding endogenous proteins and triggering immune responses in susceptible individuals.

Contact stomatitis associated with acrylic resins has been described in both prosthodontic and orthodontic settings. Clinical manifestations may include erythema, edema, discomfort, and localized mucosal inflammation.

Patients occasionally report burning sensations or subjective oral discomfort following appliance insertion. Although multifactorial in origin, chemical irritation resulting from released compounds may contribute to symptom development.

True allergy to acrylic materials is considered relatively uncommon but clinically significant. Sensitization may involve methyl methacrylate or related methacrylate derivatives.

Nickel hypersensitivity remains one of the most prevalent metal-related allergies encountered in orthodontics. For this reason, titanium-based alternatives have gained considerable interest, particularly for sensitized individuals.

Children and adolescents represent the primary recipients of removable orthodontic therapy. Because treatment often extends over several years, minimizing chronic exposure to potentially irritating substances is especially important in this population.

### 3.4. Biofilm Formation on Orthodontic Materials

Biofilm development represents a critical factor linking material science to clinical outcomes. Initial bacterial adhesion is strongly influenced by the physicochemical properties of the substrate. Surface roughness, porosity, hydrophobicity, surface energy, and wettability collectively determine the likelihood of microbial attachment. Studies comparing different orthodontic materials have demonstrated significant differences in Streptococcus mutans adhesion patterns. Rougher and more porous surfaces tend to retain larger numbers of microorganisms and facilitate maturation of the extracellular matrix.

This concept is supported by recent in vitro evidence comparing five common orthodontic materials—stainless steel, nickel–titanium, metallic brackets, ceramic brackets, and PMMA acrylic resin—which showed a clear material-dependent gradient in Streptococcus mutans colonization. In that study, PMMA exhibited the highest bacterial load and biofilm biomass, whereas stainless steel showed the lowest values; moreover, surface roughness and contact angle positively correlated with microbiological outcomes. These observations provide a strong rationale for biomimetic strategies aimed at smoothing surfaces, increasing hydrophilicity, and interfering with early bacterial adhesion [13,19].

Importantly, the adhesion interface between bacteria and material surfaces represents a key target for preventive interventions. Modifying this interface through surface engineering may reduce early colonization and disrupt subsequent biofilm development.

Biofilm accumulation may exacerbate mucosal inflammation, alter local pH, promote dysbiosis, and contribute to enamel demineralization. Consequently, reducing bacterial adhesion is increasingly recognized as a major objective of advanced orthodontic biomaterials [20].

### 3.5. Titanium and Titanium-Based Solutions

Titanium has emerged as one of the most promising biomaterials in contemporary orthodontics. Among available alloys, Ti-6Al-4V is particularly relevant because of its excellent mechanical performance, corrosion resistance, and biocompatibility. The remarkable biological behavior of titanium largely derives from its spontaneously formed titanium oxide layer. This passive film protects the underlying metal from corrosion and contributes to favorable tissue interactions. Unlike conventional nickel-containing alloys, titanium materials present substantially lower risks of nickel-related hypersensitivity reactions [21,22]. Although much of the current evidence regarding titanium surface properties originates from implantology and biomedical engineering, these findings provide valuable translational insights that may also be relevant for the development of removable orthodontic appliances. However, orthodontic-specific validation remains necessary.

Recent developments in digital orthodontics have enabled fabrication of customized titanium devices through additive manufacturing technologies such as direct metal laser sintering (DMLS). Patient-specific appliances may improve precision, reduce bulk, and enhance clinical performance while maintaining favorable biological characteristics. The translational potential of this approach is illustrated by a recent retrospective clinical study on a customized titanium Transversal Sagittal Maxillary Expander (TSME), manufactured through a digital workflow integrating intraoral scanning, CBCT, and three-dimensional facial acquisition. In a cohort of 45 growing patients, the authors reported statistically significant dento-skeletal changes after treatment, supporting the clinical feasibility of patient-specific titanium orthodontic devices. Although that study did not evaluate surface coatings, it is highly relevant to the present review because it demonstrates that digitally manufactured titanium frameworks may serve as a realistic platform for future biomimetic functionalization in interceptive orthodontics. Therefore, this study should be interpreted as evidence supporting the feasibility of customized titanium platforms rather than direct evidence for biomimetic surface functionalization [23,24].

Despite these advantages, additively manufactured titanium components require appropriate finishing and polishing procedures. Surface irregularities generated during manufacturing may otherwise promote bacterial retention and compromise tissue compatibility. Although titanium is often regarded as a highly biocompatible material, current evidence does not justify assuming automatic superiority regarding allergy prevention. Well-designed clinical studies remain necessary to confirm long-term benefits in orthodontic applications. Overall, current evidence supporting titanium-based biomimetic strategies in removable orthodontics remains limited, and many proposed applications are extrapolated from implantology and other dental disciplines.

### 3.6. Bio-Inspired Surface Modification Strategies

Bio-inspired surface engineering has emerged as a promising approach to improve the biological performance of orthodontic materials. Unlike conventional modifications that primarily alter bulk composition, bio-inspired coatings aim to regulate interactions occurring at the interface between the appliance and the oral environment. From a biomimetic perspective, the key point is the translation of a biological principle into a clinically useful material function: adhesion in wet environments, antioxidant protection, antimicrobial membrane disruption, hydrated lubrication, selective cell-material communication, or oxide-mediated passivation. Such modifications may simultaneously influence monomer diffusion, protein adsorption, bacterial adhesion, oxidative stress, and tissue compatibility (Table 1).

#### 3.6.1. Polydopamine-Based Coatings

Polydopamine (PDA) is one of the most extensively investigated bio-inspired coating materials. Its development was inspired by the remarkable adhesive capabilities of marine mussels, which use catechol-containing proteins to attach firmly to a variety of wet surfaces. The spontaneous oxidative polymerization of dopamine under mild conditions generates a thin, highly adherent coating capable of depositing on metals, polymers, ceramics, and composite materials. This universal adhesion behavior has attracted considerable interest in dental and biomedical surface engineering [25,26].

PDA is particularly attractive for orthodontic applications because it adheres strongly to PMMA, thermoplastic polymers, and metallic substrates; may function as a diffusion barrier; increases surface hydrophilicity; promotes cellular attachment and proliferation; may reduce bacterial adhesion; and provides a versatile platform for secondary functionalization. Of particular relevance to orthodontic biomaterials is the possibility that PDA may reduce the direct exposure of oral tissues to residual monomers by creating an intermediate barrier layer. Although direct evidence regarding monomer leaching reduction remains limited, several studies suggest that PDA coatings can decrease permeability and improve surface stability [27,28,29].

An additional advantage is the presence of reactive catechol and amine groups, which enable further immobilization of antimicrobial peptides, nanoparticles, growth factors, anti-inflammatory molecules, or polyphenolic compounds. Consequently, PDA is increasingly regarded as a multifunctional primer rather than merely a passive coating. Nevertheless, important challenges remain. Long-term durability under brushing, thermal cycling, enzymatic degradation, and prolonged saliva exposure has not yet been fully characterized for orthodontic applications. Although PDA coatings appear highly promising, current evidence remains largely limited to laboratory investigations. Clinical studies evaluating long-term durability, resistance to brushing, and effectiveness in orthodontic appliances are still lacking. Moreover, most currently available studies have been conducted on implant surfaces, restorative biomaterials, or general biomedical substrates rather than removable orthodontic appliances. Consequently, these findings should currently be considered translational evidence requiring orthodontic-specific validation.

#### 3.6.2. Polyphenolic Coatings

Naturally occurring polyphenols have attracted growing attention because of their antioxidant, antimicrobial, anti-inflammatory, and metal-chelating properties. Among them, tannic acid has emerged as one of the most promising candidates for surface functionalization. Tannic acid is a plant-derived polyphenol capable of forming stable interactions with proteins, polymers, and metallic surfaces through hydrogen bonding and metal coordination mechanisms [30,31,32].

Potential benefits of tannic acid-based coatings include antioxidant activity, scavenging of reactive oxygen species, reduction in oxidative stress, antimicrobial effects against oral pathogens, inhibition of biofilm formation, metal ion chelation, and relatively simple deposition procedures. From a biological perspective, oxidative stress represents one of the principal pathways implicated in methacrylate-induced cytotoxicity. Consequently, coatings possessing intrinsic antioxidant properties may contribute to mitigating adverse cellular responses.

Polyphenol-based coatings may also alter surface wettability and reduce bacterial attachment by modifying the physicochemical characteristics of the substrate. In addition, their ability to interact with metallic ions may be particularly relevant in hybrid acrylic-metal orthodontic appliances. However, concerns remain regarding coating stability, gradual degradation, discoloration potential, and maintenance of optical properties in transparent orthodontic devices. Most available evidence derives from restorative dentistry, implantology, or other biomedical applications rather than removable orthodontics. Therefore, the proposed benefits should currently be interpreted as translational rather than direct orthodontic evidence.

#### 3.6.3. Chitosan-Based Coatings

Chitosan is a naturally derived polysaccharide obtained through the deacetylation of chitin, one of the most abundant biopolymers found in nature. Because of its excellent biocompatibility, biodegradability, and antimicrobial activity, chitosan has been extensively investigated in biomedical and dental applications. Several mechanisms contribute to its biological activity, including electrostatic interaction with negatively charged bacterial membranes, disruption of membrane integrity, interference with bacterial metabolism, inhibition of biofilm development, and formation of protective barrier layers. For orthodontic materials, chitosan coatings may provide multiple advantages. Besides reducing bacterial colonization, they may act as semipermeable barriers limiting the migration of residual monomers toward the oral environment. The mucoadhesive properties of chitosan may also contribute to improved interactions with oral soft tissues, potentially reducing irritation and enhancing comfort. Despite these promising characteristics, important limitations must be considered. Chitosan exhibits pH-dependent solubility and may undergo degradation under certain environmental conditions. Long-term stability in the complex oral environment therefore remains a major area requiring further investigation. Despite encouraging antibacterial activity, coating stability under prolonged intraoral conditions remains poorly investigated [33,34]. Furthermore, most investigations have been performed using implant surfaces, restorative materials, or experimental biomaterials, whereas evidence specifically involving removable orthodontic appliances remains scarce.

#### 3.6.4. Peptide-Functionalized Surfaces

Peptide-based surface engineering represents a highly sophisticated strategy that attempts to mimic biological signaling pathways involved in host–microbe and host–material interactions. Several categories of peptides have been investigated, including antimicrobial peptides (AMPs), cell-adhesive peptides, anti-fouling peptides, and multifunctional bioactive peptides. Antimicrobial peptides can selectively disrupt bacterial membranes while exhibiting relatively limited toxicity toward mammalian cells. This selective mechanism may offer advantages over conventional antimicrobial agents that rely on non-specific cytotoxic effects. Potential benefits include reduced bacterial adhesion, inhibition of biofilm maturation, preservation of oral microbial balance, and improved compatibility with oral tissues. Peptides promoting cellular adhesion may also support favorable interactions between appliances and mucosal tissues, potentially reducing inflammatory responses. However, peptide-based systems face significant challenges, including enzymatic degradation by saliva, limited long-term stability, complex manufacturing procedures, and elevated production costs. Consequently, their routine clinical implementation remains limited despite considerable scientific interest. Current evidence is mainly experimental and largely derives from implantology and general biomaterials research rather than orthodontic applications [35,36].

#### 3.6.5. Biomimetic Hydrogel-like Barriers

Hydrogel-inspired coatings seek to replicate the hydrated nature of biological interfaces such as oral mucosa and salivary pellicles. These systems create water-rich surface layers capable of modifying interactions between biomaterials and the surrounding environment. The main mechanisms include formation of highly hydrated barriers, reduction in protein adsorption, inhibition of bacterial attachment, decreased friction, improved patient comfort, and limitation of molecular diffusion. Because bacterial colonization often begins with protein adsorption onto biomaterial surfaces, reducing protein deposition may indirectly impair subsequent microbial adhesion. Furthermore, hydrated coatings may act as diffusion barriers that slow the migration of residual monomers from the polymer matrix toward oral tissues. The concept is particularly attractive for removable orthodontic appliances because patient comfort, mucosal tolerance, and biofilm control are all influenced by surface hydration characteristics. However, hydrogel-like coatings frequently encounter durability challenges under repeated mechanical loading and cleaning procedures. To date, most available evidence has been obtained from biomedical engineering and implantology, with only limited application to removable orthodontic materials [37,38].

#### 3.6.6. Titanium Dioxide- and Oxide-Based Surfaces

Titanium dioxide (TiO_2_) and related oxide-based modifications constitute one of the most extensively investigated classes of functional surfaces in dentistry and biomedical engineering. The favorable biological performance of titanium largely derives from its oxide layer, which provides corrosion resistance, chemical stability, enhanced biocompatibility, and reduced ion release. Additional modifications of titanium oxide surfaces may further improve their functional properties. Potential benefits include antimicrobial activity, photocatalytic effects, anti-corrosion behavior, improved wettability, and enhanced tissue compatibility. Under appropriate conditions, TiO_2_ can generate reactive oxygen species capable of damaging microbial cells and disrupting biofilm formation. This feature has generated considerable interest for applications requiring long-term bacterial control. Anodization procedures may also modify oxide layer thickness, nanotopography, and surface energy, potentially influencing both bacterial and cellular responses. Despite these advantages, concerns remain regarding the long-term behavior of photocatalytic systems within the oral environment. The balance between antimicrobial efficacy and host tissue safety requires careful evaluation before widespread clinical implementation. Additional long-term clinical studies are required before routine orthodontic application can be recommended. Most published evidence concerns implant surfaces rather than orthodontic appliances; therefore, direct translation into removable orthodontics should be interpreted cautiously [39,40].

#### 3.6.7. Nanoparticle-Enriched Coatings

Nanotechnology has introduced additional possibilities for controlling microbial colonization and improving material performance. Nanoparticles incorporated into surface coatings include silver nanoparticles, zinc oxide nanoparticles, copper nanoparticles, bioactive glass nanoparticles, and fluoride-releasing nanostructures. Although some nanoparticle systems may be combined with bio-inspired approaches, it is important to distinguish nano-engineered antibacterial coatings from genuinely bio-inspired surface modifications. Silver nanoparticles are among the most extensively studied because of their broad-spectrum antimicrobial activity. Zinc oxide nanoparticles have similarly demonstrated antibacterial properties together with relatively favorable biocompatibility profiles. Potential benefits include reduced bacterial growth, inhibition of biofilm maturation, improved surface antimicrobial properties, and potential synergistic effects with other coatings. However, concerns regarding nanoparticle release, long-term toxicity, environmental impact, and regulatory approval remain significant. Consequently, nanoparticle-based coatings should be considered promising but still incompletely validated solutions for orthodontic applications. In addition, much of the available literature originates from restorative dentistry, implantology, or general biomaterials research rather than orthodontic devices [41,42].

### 3.7. Non-Bio-Inspired Barrier Coatings as Comparators

Although bio-inspired coatings have attracted substantial interest, several non-bio-inspired surface treatments have also been investigated to improve the biological performance of dental polymers. Examples include polyurethane coatings, silicone-based coatings, fluoropolymer coatings, plasma-polymerized films, commercial protective varnishes, and synthetic diffusion barriers. These systems generally function through purely physical mechanisms rather than biological mimicry. Their principal objectives include reducing surface permeability, decreasing water absorption, limiting monomer diffusion, improving chemical stability, and increasing resistance to environmental degradation. In several studies, synthetic barrier coatings have demonstrated measurable reductions in the release of residual compounds. However, unlike bio-inspired approaches, they typically lack intrinsic antimicrobial, antioxidant, anti-inflammatory, or tissue-interactive properties. As a result, their biological benefits are often restricted to passive barrier effects. The comparison between bio-inspired and conventional coatings highlights a fundamental difference in design philosophy. Traditional barrier systems primarily attempt to isolate the material from the oral environment, whereas bio-inspired coatings actively seek to regulate biological interactions occurring at the material–tissue interface. This distinction is particularly relevant when considering the multifactorial nature of orthodontic biomaterial-associated complications, which involve not only monomer release but also biofilm formation, inflammatory signaling, oxidative stress, and host tissue responses. Overall, current evidence suggests that multifunctional bio-inspired coatings may offer broader biological advantages than conventional barrier systems. Nevertheless, direct comparative studies remain scarce, and definitive conclusions regarding clinical superiority cannot yet be drawn. However, most comparative studies have not been specifically performed on removable orthodontic appliances, limiting direct clinical extrapolation [43,44].

## 4. Proposed Mechanisms of Action

The potential benefits of bio-inspired surface modifications are not attributable to a single mechanism. Rather, these coatings may simultaneously influence multiple physicochemical and biological processes occurring at the interface between orthodontic materials and the oral environment. Based predominantly on experimental and translational evidence available in implantology, restorative dentistry, and biomaterials research, the proposed effects can be broadly categorized into five principal mechanisms.

### 4.1. Physical Barrier Effect

One of the most intuitive mechanisms is the formation of a physical barrier capable of reducing the diffusion of residual monomers, degradation products, and potentially harmful compounds from the bulk material toward surrounding tissues.

Residual monomers are typically released through diffusion pathways facilitated by water sorption and polymer network permeability. Experimental studies suggest that surface coatings may partially obstruct these pathways, thereby decreasing the rate and magnitude of leaching. The effectiveness of this mechanism depends on several factors, including coating thickness, coating continuity, adhesion to the substrate, resistance to mechanical degradation, and permeability characteristics. Experimental evidence suggests that polydopamine, hydrogel-like coatings, polymeric barrier layers, and some nanoparticle-containing systems may contribute to limiting molecular diffusion. Importantly, even modest reductions in cumulative monomer exposure may become clinically relevant during prolonged orthodontic treatment, particularly in pediatric patients who wear removable appliances for several years.

### 4.2. Chemical Binding and Neutralization

Certain bio-inspired coatings possess chemically active functional groups capable of interacting with reactive compounds released from orthodontic materials. Polydopamine contains catechol and amine groups, while polyphenolic coatings contain hydroxyl-rich structures capable of participating in hydrogen bonding, redox reactions, and metal chelation. Potential consequences include reduced free reactive species, sequestration of metallic ions, modulation of oxidative pathways, and decreased bioavailability of potentially harmful molecules. Polyphenolic coatings may be particularly effective because tannins and related compounds possess strong antioxidant and chelating properties. These characteristics could theoretically reduce biological exposure not only to resin-derived compounds but also to ions released from metallic components. Most mechanistic evidence derives from studies performed on implant surfaces and restorative biomaterials. Although the magnitude of these effects remains incompletely characterized, such mechanisms represent a unique advantage over conventional passive barrier coatings.

### 4.3. Modulation of Surface Energy and Wettability

Surface energy plays a critical role in determining how proteins, bacteria, and host cells interact with biomaterials. The earliest stage of biofilm formation involves adsorption of salivary proteins, which subsequently mediate bacterial attachment. Consequently, modifications affecting surface wettability may significantly influence downstream biological events. Bio-inspired coatings frequently increase surface hydrophilicity through the introduction of polar functional groups. Potential consequences include reduced hydrophobic bacterial interactions, altered protein adsorption patterns, decreased initial bacterial adhesion, and improved compatibility with host tissues. Polydopamine-coated surfaces often exhibit increased wettability compared with untreated polymers. Similar effects have been reported for hydrogel-inspired coatings and several peptide-functionalized systems. These observations originate predominantly from experimental surface-science investigations rather than orthodontic clinical studies. These modifications may be particularly important because they directly influence the adhesive interface between the orthodontic appliance and the oral environment—a key consideration highlighted by recent interest in material–biofilm interactions.

### 4.4. Antioxidant and Anti-Inflammatory Effects

A growing body of evidence suggests that oxidative stress contributes significantly to methacrylate-associated cytotoxicity. Reactive oxygen species generated following monomer exposure can activate inflammatory pathways, impair cellular function, and induce DNA damage. Several bio-inspired coatings possess intrinsic antioxidant properties capable of counteracting these mechanisms. Polyphenolic systems are particularly relevant because they can scavenge reactive oxygen species, reduce oxidative stress, modulate inflammatory signaling, and protect cellular structures from oxidative damage. Similarly, some peptide-based and chitosan-based systems may influence inflammatory responses through interactions with immune pathways and cellular signaling mechanisms. To date, evidence supporting these mechanisms remains largely experimental. Although direct clinical evidence remains limited, the possibility of reducing inflammation through surface engineering represents one of the most attractive aspects of bio-inspired approaches.

### 4.5. Antibacterial and Anti-Biofilm Activity

Control of microbial colonization represents one of the most consistently reported benefits of advanced surface modifications. The development of biofilm on orthodontic appliances involves multiple stages: protein adsorption, initial bacterial attachment, bacterial proliferation, extracellular matrix production, and biofilm maturation. Bio-inspired coatings may interfere with one or more of these stages. Potential mechanisms include reduction in bacterial adhesion, disruption of bacterial membranes, inhibition of quorum sensing, prevention of extracellular matrix formation, and modification of local surface chemistry. Chitosan coatings primarily act through membrane interactions, whereas antimicrobial peptides exert selective bactericidal effects. Polydopamine-based systems frequently serve as carriers for antimicrobial molecules, while TiO_2_-based surfaces may exert photocatalytic antibacterial activity. Although supported mainly by in vitro evidence, anti-biofilm effects may indirectly reduce inflammatory stimulation and improve oral health during orthodontic treatment.

## 5. Clinical Implications

Children and adolescents represent the population most frequently exposed to removable orthodontic appliances. Treatment often extends over prolonged periods, increasing cumulative exposure to residual monomers, degradation products, and microbial biofilms. In this context, even modest improvements in material biocompatibility may acquire significant clinical relevance. Surface-engineered appliances may eventually reduce irritation, bacterial accumulation, and inflammatory responses, thereby potentially improving patient comfort and compliance if these benefits are confirmed in well-designed prospective clinical studies. Furthermore, minimizing biological risks is particularly important in younger patients because oral tissues may be exposed continuously throughout critical developmental stages. Patients presenting previous allergic reactions to methacrylates, acrylic resins, or metallic components represent a subgroup that may particularly benefit from advanced biomaterials. Potential strategies include reducing residual monomer release through barrier coatings, using bio-inspired anti-inflammatory surfaces, replacing nickel-containing alloys with titanium-based alternatives, and implementing hybrid approaches that combine material substitution with surface engineering. Although preliminary findings are encouraging, current evidence remains predominantly preclinical. Consequently, these approaches should currently be regarded as promising experimental strategies whose clinical effectiveness requires confirmation through prospective clinical trials.

The most immediate applications of bio-inspired coatings involve removable orthodontic devices, including acrylic plates, Hawley retainers, functional appliances, removable expanders, clear aligners, and vacuum-formed retainers. Because these devices remain in prolonged contact with oral mucosa and saliva, they represent attractive candidates for future evaluation of surface modification technologies. Additionally, removable appliances allow easier replacement and maintenance compared with fixed devices, potentially facilitating future implementation of coated materials. Many contemporary orthodontic appliances combine polymeric and metallic components within the same structure. Examples include acrylic plates with stainless steel clasps, functional appliances incorporating metal frameworks, removable expansion systems, and digitally manufactured hybrid devices. In such situations, both residual monomer release and metallic ion release may contribute to biological responses. Consequently, integrated strategies simultaneously targeting polymeric and metallic interfaces may offer the greatest clinical benefit. Titanium-based frameworks combined with bio-inspired coatings may represent a promising future research direction; however, their routine clinical application will require robust long-term clinical validation.

## 6. Limitations of the Current Evidence and Future Perspectives

Despite the growing interest in bio-inspired surface engineering for dental and orthodontic applications, the current body of evidence presents several important limitations that should be carefully considered when interpreting available findings. Most studies investigating monomer release, biological effects, and surface modifications have been conducted under laboratory conditions. In vitro experiments provide valuable mechanistic insights and allow strict control of experimental variables; however, they cannot fully reproduce the complexity of the oral environment. Clinical exposure involves multiple interacting factors, including salivary flow, dietary habits, oral hygiene procedures, microbial ecology, host immune responses, and individual variations in appliance wear. Consequently, laboratory observations may either overestimate or underestimate actual clinical effects. Furthermore, exposure conditions used in cell culture experiments frequently involve concentrations and durations that differ substantially from those encountered intraorally. A major limitation of the literature is the absence of standardized aging methodologies. Studies evaluating monomer release or coating performance employ highly variable protocols, including differences in storage media, duration of immersion, temperature conditions, thermal cycling procedures, pH cycling, brushing simulation, and mechanical loading. As a result, direct comparison among studies is often difficult. The development of standardized testing protocols would significantly improve reproducibility and facilitate comparison of emerging technologies. Although many bio-inspired coatings demonstrate promising short-term results, relatively little information is available regarding their long-term stability. Orthodontic appliances may remain in service for months or years, requiring coatings to withstand repeated cleaning procedures, salivary enzymes, mechanical wear, thermal fluctuations, dietary acids, and microbial colonization. The durability of the coating–substrate interface remains one of the most critical determinants of clinical success. Particularly for peptide-based systems, chitosan coatings, and hydrogel-like barriers, long-term retention of biological functionality remains insufficiently documented. Orthodontic materials are highly heterogeneous. Differences may involve polymer composition, monomer content, degree of conversion, manufacturing procedures, additive technologies, polishing protocols, and post-processing treatments. Consequently, results obtained using one material cannot necessarily be generalized to other orthodontic systems. Similarly, data derived from restorative composites or prosthodontic resins may not accurately reflect the behavior of removable orthodontic appliances. Most studies evaluate surrogate outcomes such as bacterial counts, contact angle measurements, surface roughness, cell viability, and monomer release. While these parameters are scientifically valuable, relatively few investigations assess clinically meaningful endpoints. In particular, there is a notable lack of evidence regarding reduction in allergic reactions, prevention of contact stomatitis, improvement of patient-reported outcomes, long-term mucosal tolerance, and incidence of oral lesions. Consequently, the true clinical impact of bio-inspired coatings remains incompletely established. Only a small number of investigations directly compare coated and uncoated orthodontic appliances under clinically relevant conditions. Furthermore, head-to-head comparisons between different coating technologies are rare. As a result, it remains difficult to determine which surface engineering strategy offers the most favorable balance between biological performance, durability, cost, and manufacturability. Many bio-inspired coatings have been developed primarily for implantology, restorative dentistry, or biomedical devices rather than orthodontics. Although these studies provide valuable conceptual foundations, orthodontic appliances exhibit unique characteristics, including prolonged exposure to saliva, removable use patterns, repeated insertion and removal, extensive plaque accumulation surfaces, and frequent mechanical cleaning. Therefore, extrapolation from other dental disciplines should be approached cautiously. Given that children and adolescents constitute the primary population receiving removable orthodontic treatment, the scarcity of long-term pediatric data represents a significant gap in knowledge. Future investigations should specifically address age-related differences in oral ecology, appliance wear patterns, and biological responses to prolonged biomaterial exposure. Overall, the lack of standardized testing methodologies, the predominance of laboratory investigations, limited long-term durability data, and the scarcity of well-designed clinical trials currently represent the principal barriers preventing routine clinical implementation of bio-inspired surface modifications in removable orthodontics.

Future research should primarily focus on generating high-quality evidence supporting the clinical translation of bio-inspired surface modifications for removable orthodontic appliances. Several research directions appear particularly promising. Future studies should adopt harmonized protocols incorporating artificial saliva, dynamic pH cycling, thermal cycling, brushing simulation, and mechanical loading. The adoption of standardized ISO-compliant testing methodologies would substantially improve reproducibility and facilitate comparisons among different coating technologies. Such approaches would improve the clinical relevance of laboratory investigations and facilitate comparisons among studies. Extended evaluations are needed to characterize cumulative monomer release throughout the entire lifespan of orthodontic appliances. Future investigations should also include long-term aging protocols reproducing clinically relevant conditions, including thermal cycling, brushing simulation, enzymatic degradation, and prolonged saliva exposure. Particular attention should be given to aged materials, repeated appliance use, degradation products, and interactions with oral biofilms. These investigations may help clarify the relationship between chronic low-level exposure and long-term biological outcomes. Traditional monoculture systems provide limited representation of oral tissues. Future research should increasingly employ oral keratinocytes, gingival fibroblasts, immune-cell co-cultures, three-dimensional oral mucosa models, and organotypic tissue constructs. Such systems may provide a more realistic assessment of tissue responses to coated materials.

Many current studies focus primarily on Streptococcus mutans. However, oral biofilms consist of highly complex microbial communities; therefore, future investigations should also evaluate multi-species biofilms, dysbiosis-associated communities, fungal–bacterial interactions, and biofilm maturation dynamics. These models may better reflect real clinical conditions. The transition from laboratory research to clinical validation represents the most important future challenge. Potential approaches include intraoral splint models, short-term appliance studies, prospective clinical trials, patient-reported outcome assessments, and hypersensitivity monitoring. Such investigations are essential for determining whether laboratory improvements translate into meaningful clinical benefits. Future surface engineering strategies will likely combine multiple biological functions within a single coating. These hybrid systems may simultaneously provide barrier, antimicrobial, antioxidant, and anti-inflammatory effects. Future coatings may incorporate bioactive components capable of supporting enamel protection. Potential functions include fluoride release, calcium-phosphate delivery, remineralization promotion, and reduction in white spot lesion risk. Such approaches may further expand the preventive role of orthodontic appliances. The integration of additive manufacturing and digital workflows offers unprecedented opportunities for personalized orthodontics [45]. Digital manufacturing may facilitate the future integration of biomimetic coatings into customized titanium orthodontic appliances, although additional clinical studies will be required to demonstrate the long-term benefits of these combined approaches. Future developments may combine customized titanium frameworks with bio-inspired surface functionalization, creating highly individualized therapeutic devices [46]. Clinical experience with customized titanium appliances already suggests that digital manufacturing can improve workflow standardization and patient-specific adaptation. Consequently, the next logical step may be the integration of biomimetic coatings into these digitally manufactured devices in order to unite structural customization with optimized biological performance [23,24]. Artificial intelligence and computational modeling may support coating optimization and experimental design; however, their clinical value will ultimately depend on validation through standardized laboratory and clinical investigations [47,48,49,50]. Potential applications include predicting bacterial adhesion, optimizing surface topography, simulating monomer diffusion, and identifying optimal coating combinations. These technologies may accelerate the development of next-generation orthodontic biomaterials [51,52,53,54,55].

## 7. Discussion

The present review highlights the growing recognition that biological safety in orthodontics extends beyond traditional considerations of mechanical performance and treatment efficacy. Removable orthodontic appliances are continuously exposed to a complex oral environment that may promote material degradation, monomer release, ion release, microbial colonization, and inflammatory responses. Consequently, strategies aimed at improving the biological profile of orthodontic materials have become increasingly relevant. The available evidence suggests that bio-inspired surface engineering represents a promising experimental approach whose clinical value remains to be established; however, its clinical effectiveness remains to be confirmed through well-designed prospective clinical studies. Rather than replacing established orthodontic materials, surface modifications seek to optimize their interactions with oral tissues and microbial communities.

In this context, coatings can be viewed as biological interfaces that mediate communication between the material and the host environment.

A central finding emerging from the literature is the interconnected nature of three major research domains: material safety, surface adhesion phenomena, and titanium-based technologies. The first pillar concerns the recognition that orthodontic resins are not biologically inert. Although modern materials are generally considered safe, residual monomers, degradation products, and associated oxidative mechanisms may influence cellular responses, inflammatory pathways, and microbial ecology. This does not imply that contemporary orthodontic materials are unsafe; rather, it emphasizes the importance of continuous efforts to optimize their biological performance. The second pillar involves the concept of the adhesive interface. Virtually all biological interactions occur at the material surface. Protein adsorption, bacterial attachment, cellular adhesion, inflammatory signaling, and molecular diffusion are governed by events occurring within the first few nanometers of the biomaterial interface. Consequently, surface properties such as roughness, wettability, surface energy, porosity, and chemical functionality may profoundly influence clinical outcomes. This emphasis on adhesion and adhesive interfaces is particularly relevant because the interface represents the common denominator linking monomer release, bacterial colonization, and tissue compatibility. From this perspective, bio-inspired coatings may be interpreted as tools for engineering a more favorable adhesive interface rather than merely adding a protective layer. The third pillar involves the increasing role of titanium and digitally manufactured orthodontic devices. Current evidence suggests that titanium offers several advantages, including excellent corrosion resistance, stable oxide formation, favorable biocompatibility, reduced relevance of nickel hypersensitivity, and compatibility with additive manufacturing. The emergence of patient-specific titanium appliances further expands opportunities for individualized treatment approaches. Importantly, titanium should not be viewed as a competing strategy to bio-inspired coatings. Rather, both approaches may be complementary. Future orthodontic devices may combine titanium-based frameworks with advanced surface functionalization technologies to achieve optimal biological performance. An ideal orthodontic coating must satisfy multiple requirements simultaneously. Beyond reducing monomer release and bacterial adhesion, it should preserve mechanical strength, dimensional stability, appliance fit, transparency when required, polishability, patient comfort, affordability, and ease of manufacturing. Achieving all these objectives simultaneously remains challenging. For example, coatings that improve antimicrobial activity may alter transparency, while highly hydrated surfaces may exhibit reduced mechanical durability. Therefore, future development should focus on balancing biological benefits with practical clinical requirements. Although all bio-inspired surface modification strategies share the common objective of improving the biological performance of orthodontic materials, important differences exist regarding their mechanisms of action, level of evidence, and translational potential. Polydopamine represents one of the most versatile approaches because it combines excellent substrate adhesion with the possibility of secondary functionalization using antimicrobial peptides, antioxidants, or bioactive molecules. However, evidence supporting its long-term stability under clinically relevant orthodontic conditions remains limited. Polyphenolic coatings primarily provide antioxidant and anti-inflammatory effects and may additionally reduce bacterial adhesion, although concerns regarding discoloration and optical stability remain unresolved. Chitosan-based coatings exhibit promising antimicrobial and mucoadhesive properties, but their durability is influenced by pH-dependent degradation. Peptide-functionalized surfaces offer highly selective biological activity but are limited by enzymatic degradation, manufacturing complexity, and elevated costs. Hydrogel-like coatings may improve lubrication and reduce protein adsorption; however, their long-term mechanical stability remains uncertain. Finally, titanium dioxide-based surfaces demonstrate favorable corrosion resistance and antimicrobial potential, although most available evidence derives from implantology rather than removable orthodontics. Overall, no single strategy currently fulfills all the biological, mechanical, and clinical requirements required for routine orthodontic use. Future developments will likely involve multifunctional coatings integrating complementary mechanisms within a single surface-engineering platform.

Despite encouraging findings, caution is warranted when interpreting current evidence. Many published studies demonstrate promising reductions in bacterial adhesion, improvements in wettability, and enhanced cellular responses. However, robust clinical evidence demonstrating prevention of allergic reactions, contact stomatitis, or other clinically significant adverse outcomes remains limited. Similarly, direct evidence showing substantial reductions in long-term monomer exposure following coating application is still relatively scarce. Consequently, bio-inspired surface engineering should currently be regarded as a promising experimental field rather than an established clinical strategy. Although laboratory findings are encouraging, additional standardized in situ and clinical investigations are required before routine implementation can be recommended. The available evidence supports continued investigation and technological development, but definitive conclusions regarding routine clinical implementation cannot yet be drawn. Future well-designed in situ and clinical studies will be essential to determine whether the biological advantages observed in laboratory settings translate into meaningful improvements in patient care.

## 8. Conclusions

Removable orthodontic appliances are widely used for prolonged periods and are continuously exposed to saliva, microbial biofilms, thermal fluctuations, and mechanical stresses. Although current resin-based and metallic orthodontic materials are generally considered clinically safe, residual monomers, degradation products, and metallic ions may contribute to cytotoxic, inflammatory, and hypersensitivity-related responses.

The evidence reviewed indicates that bio-inspired surface modifications have considerable potential to improve the biological safety of removable orthodontic appliances. By engineering the material–tissue interface, these coatings may reduce monomer diffusion, improve surface wettability, decrease bacterial adhesion and biofilm formation, attenuate oxidative and inflammatory responses, and enhance biocompatibility. Among the most promising approaches are polydopamine-based coatings, polyphenolic systems, chitosan-derived coatings, peptide-functionalized surfaces, biomimetic hydrogel-like barriers, and titanium oxide-based modifications.

The literature also highlights the importance of surface and adhesive interfaces as key determinants of biological performance. In parallel, titanium and digitally manufactured titanium-based appliances offer additional opportunities for improving biocompatibility, particularly in patients with metal hypersensitivity.

However, current evidence remains predominantly based on in vitro studies, and significant gaps persist regarding long-term durability, clinical effectiveness, and the actual reduction in hypersensitivity-related complications. Future research should focus on standardized aging protocols, multi-species biofilm models, and well-designed clinical studies to validate these technologies under realistic oral conditions.

Overall, bio-inspired surface modifications represent a promising direction for improving the biological safety of removable orthodontic appliances. However, current evidence remains predominantly preclinical, and well-designed standardized clinical studies with long-term follow-up are required before routine clinical implementation can be recommended. Future investigations should also evaluate coating durability under clinically relevant aging conditions, patient-reported outcomes, and compatibility with contemporary digital manufacturing workflows.

## Figures and Tables

**Table 1:** Biomimetic rationale and translational relevance of the main surface modification strategies discussed in this review.

Strategy	Biological Inspiration/Principle	Expected Orthodontic Function	Main Translational Limitation
Polydopamine	Mussel-inspired catechol adhesion in wet environments	Universal primer, diffusion barrier, increased hydrophilicity, platform for secondary functionalization	Long-term stability under brushing, saliva, enzymes, and thermal cycling
Tannic acid/polyphenols	Plant-derived antioxidant and metal-chelating systems	Reactive oxygen species scavenging, anti-inflammatory potential, antimicrobial activity, ion chelation	Possible discoloration and incomplete data on optical stability
Chitosan	Natural cationic polysaccharide with antimicrobial and mucoadhesive properties	Biofilm reduction, semipermeable barrier effect, improved soft-tissue interaction	pH-dependent solubility and uncertain durability
Antimicrobial or bioactive peptides	Host-defense peptides and cell-signaling motifs	Selective anti-biofilm action and controlled cell-material interactions	Enzymatic degradation, cost, and manufacturing complexity
Hydrogel-like coatings	Hydrated mucosal and salivary pellicle-like interfaces	Reduced friction, protein adsorption control, improved comfort, limited molecular diffusion	Mechanical wear and retention under repeated cleaning
Titanium dioxide/oxide layers	Stable mineralized oxide interfaces and passivation	Corrosion resistance, improved wettability, reduced ion release, antimicrobial potential	Need to balance photocatalytic activity and host–cell safety
Nanoparticle-enriched hybrid coatings	Nano-scale antimicrobial or remineralizing effects inspired by biological mineral interfaces	Biofilm control, possible remineralizing or fluoride-releasing functions	Potential nanoparticle release, toxicity, and regulatory concerns
Conventional barrier coatings	Non-biomimetic comparator strategy	Physical reduction in water sorption and monomer diffusion	Usually lack intrinsic antimicrobial, antioxidant, or host-interactive functions

## Data Availability

No new data were created or analyzed in this study. Data sharing is not applicable to this article.
